# Supplementary material for: An open-source framework for large-scale, flexible evaluation of biomedical text mining systems
Source: J Biomed Discov Collab. 2008 Jan 29;3:1. doi: 10.1186/1747-5333-3-1 (PMC2276192; doi:10.1186/1747-5333-3-1)
Supplement: Additional file 1 — Evaluation platform source code. The additional file contains the software evaluation framework discussed in this paper. The most current release can be downloaded from SourceForge.net [1]. See the accompanying README file for instructions on installation and use. Also included in the distribution is a collection of UIMA wrappers for some commonly used BioNLP tools and annotated corpora. [file 1747-5333-3-1-S1.zip › bionlp_uima_repository-1.0.1/docs/api/src-html/edu/uchsc/ccp/uima/ae/nlp/ei/gene/ABNER_AE.html]

```
001    /*
002     *
003     * The Original Code is UCHSC UIMA.
004     *
005     * The Initial Developer of the Original Code is University of Colorado.  
006     * Copyright (C) 2005.  All Rights Reserved.
007     *
008     * UCHSC Utilities was developed by the Center for Computational Pharmacology
009     * (http://compbio.uchsc.edu) at the University of Colorado Health 
010     *  Sciences Center School of Medicine with support from the National 
011     *  Library of Medicine.  
012     *
013     *
014     * Contributor(s):
015     *   William A. Baumgartner <william.baumgartner@uchsc.edu> (Original Author)
016     *   
017     * Created: August 2005
018     * Dependencies:
019     * ToDo: 
020     *      This class should probably use the findSpans method in GenericTextUtils
021     * Changes:
022     *      03/10/06 - wab - Puntation (periods) are now replaced by " ." (notice the space) in the input text before searching for gene names. 
023     *                       This is in response to Jim's request because ABNER was not able to find "NF-kappa B." at 
024     *                       the end of a sentence.
025     *      02/15/06 - rjf - Added annotation back-pointer into mentions created by
026     *                        ABNER annotator
027     *      01/09/06 - wab - Made necessary changes to conform with revised Type System
028     *                     - Specifically, the new ClassMention structure
029     *      
030     */
031    package edu.uchsc.ccp.uima.ae.nlp.ei.gene;
032    
033    import java.util.HashMap;
034    import java.util.regex.Matcher;
035    import java.util.regex.Pattern;
036    
037    import org.apache.uima.analysis_engine.ResultSpecification;
038    import org.apache.uima.analysis_engine.annotator.AnnotatorConfigurationException;
039    import org.apache.uima.analysis_engine.annotator.AnnotatorContext;
040    import org.apache.uima.analysis_engine.annotator.AnnotatorContextException;
041    import org.apache.uima.analysis_engine.annotator.AnnotatorInitializationException;
042    import org.apache.uima.analysis_engine.annotator.AnnotatorProcessException;
043    import org.apache.uima.analysis_engine.annotator.JTextAnnotator_ImplBase;
044    import org.apache.uima.jcas.JCas;
045    import org.apache.uima.jcas.cas.FSArray;
046    
047    import abner.Tagger;
048    import edu.uchsc.ccp.uima.annotation.CCPAnnotationSet;
049    import edu.uchsc.ccp.uima.annotation.CCPAnnotator;
050    import edu.uchsc.ccp.uima.annotation.CCPSemanticAnnotation;
051    import edu.uchsc.ccp.uima.annotation.CCPSpan;
052    import edu.uchsc.ccp.uima.mention.CCPClassMention;
053    import edu.uchsc.ccp.util.nlp.mention.ClassMentionTypes;
054    
055    /**
056     * The class incorporates the ABNER entity identification system into the UIMA analysis engine framework. This class outputs 5 distinct
057     * annotation types. In the CCPTypeSytem, all five types fall under the SemanticAnnotation classification.
058     * 
059     * @author William A. Baumgartner, Jr.
060     * 
061     */
062    public class ABNER_AE extends JTextAnnotator_ImplBase {
063        private final boolean DEBUG = false;
064    
065        Tagger abnerTagger = null;
066    
067        private int startLookingForMatchesAt = 0;
068    
069         public static final String PARAM_USE_BIOCREATIVE_MODEL = "UseBioCreativeModel";
070         boolean useBioCreativeModel = false;
071         
072         public void initialize(AnnotatorContext ac) throws AnnotatorInitializationException, AnnotatorConfigurationException {
073    
074            
075             try {
076                 useBioCreativeModel = ((Boolean) ac.getConfigParameterValue(PARAM_USE_BIOCREATIVE_MODEL)).booleanValue();
077             } catch (AnnotatorContextException e) {
078                 e.printStackTrace();
079             }
080    
081             // initialize ABNER tagger
082             if (useBioCreativeModel) {
083                 abnerTagger = new Tagger(Tagger.BIOCREATIVE);
084             } else {
085                 abnerTagger = new Tagger(Tagger.NLPBA);
086             }
087    
088             // turn on ABNER tokenization
089             abnerTagger.setTokenization(true);
090    
091             super.initialize(ac);
092         }
093         
094        // private int annotationSetID;
095    
096        /*
097         * (non-Javadoc)
098         * 
099         * @see com.ibm.uima.analysis_engine.annotator.JTextAnnotator#process(com.ibm.uima.jcas.impl.JCas,
100         *      com.ibm.uima.analysis_engine.ResultSpecification)
101         */
102        /**
103         * This method employs the ABNER entity identification system to search the document text for Gene, RNA, DNA, Cell Line, and Cell Type
104         * mentions.
105         */
106        public void process(JCas jcas, ResultSpecification arg1) throws AnnotatorProcessException {
107            // hashmap to keep track of annotations that have already been stored...
108            // this prevents duplicate annotations from being stored.
109            HashMap<String, String> annotMap = new HashMap<String, String>();
110    
111            // get document text
112            String docText = jcas.getDocumentText();
113    
114    ////         get the document-level annotations
115    //         String docID;
116    //         int documentCollectionID = -1;
117    //         Iterator it = jcas.getJFSIndexRepository().getAnnotationIndex(CCPDocumentInformation.type).iterator();
118    //         if (it.hasNext()) { /* there will be at most one CCPDocumentInformation annotation */
119    //         CCPDocumentInformation docInfo = (CCPDocumentInformation) it.next();
120    //         docID = docInfo.getDocumentID();
121    //         documentCollectionID = docInfo.getDocumentCollectionID();
122    //         }
123    
124    //        // get the document section IDs and lengths
125    //       Iterator it = jcas.getJFSIndexRepository().getAnnotationIndex(CCPDocumentSection.type).iterator();
126    //        ArrayList<int[]> sections = new ArrayList<int[]>(); // used for
127    //        // temporary storage
128    //        // of
129    //        // section id and length
130    //        // information
131    //        while (it.hasNext()) { // there will be at most one
132    //            // CCPDocumentInformation annotation
133    //            CCPDocumentSection docSection = (CCPDocumentSection) it.next();
134    //            int[] section = new int[2];
135    //            section[0] = docSection.getSectionID();
136    //            section[1] = docSection.getSectionLength();
137    //            sections.add(section);
138    //        }
139    //        int[] sectionIDs = new int[sections.size()];
140    //        int[] sectionLengths = new int[sections.size()];
141    //        for (int i = 0; i < sections.size(); i++) {
142    //            int[] section = (int[]) sections.get(0);
143    //            sectionIDs[i] = section[0];
144    //            sectionLengths[i] = section[1];
145    //        }
146    
147            // find entities in docText
148            /*
149             * 03/10/06 - wab - ABNER does not see genes that end a sentence, or at least does not recognize "NF-kappa B." When that ends a
150             * sentence. So, we will see if it does better if we remove periods before searching for gene names.
151             */
152            //String docTextWithoutPunctuation = docText.replaceAll("\\.", " \\.");
153            String docTextWithoutPunctuation = docText;
154    
155            String[][] entities = abnerTagger.getEntities(docTextWithoutPunctuation);
156    
157            if (DEBUG) {
158                System.out.println("---------------------  DOCTEXT  --------------------------");
159                System.out.println(docText);
160                System.out.println("DOC TEXT WITHOUT PUNCTUATION: " + docTextWithoutPunctuation);
161                System.out.println("----------------------------------------------------------");
162                for (int i = 0; i < entities[0].length; i++) {
163                    System.out.println("i: " + i + " element: " + entities[0][i] + "\ttag: " + entities[1][i]);
164                }
165                System.err.println("ABNER has detected " + entities[0].length + " entities.");
166            }
167    
168            /*
169             * This variable checks to make sure that we create an annotation for every entity found.
170             */
171            int annotationCount = 0;
172    
173            startLookingForMatchesAt = 0;
174            for (int i = 0; i < entities[0].length; i++) {
175    
176                String entity = entities[0][i];
177    
178                if (DEBUG) {
179                    System.err.println("ABNER is looking for " + entities[0][i] + " which is a " + entities[1][i]);
180                }
181                // find start/end indexes
182                String[] span = findSpan(entity, docText);
183    
184                if (span[0] != null) {
185    //                // determine document section
186    //                int documentSectionID = -1;
187    //                try {
188    //                    documentSectionID = GenericTextUtils.determineDocumentSection(Integer.parseInt(span[0]), sectionIDs, sectionLengths);
189    //                } catch (Exception e) {
190    //                    documentSectionID = 0;
191    //                }
192    
193                    String key = span[0] + " " + span[1] + " " + entities[1][i];
194                    if (!annotMap.containsKey(key)) { // make certain no duplicate
195                        // annotations are stored
196                        annotMap.put(key, "");
197    
198                        String mentionName = "";
199                        if (entities[1][i].equalsIgnoreCase("protein")) {
200                            mentionName = ClassMentionTypes.PROTEIN;
201                        } else if (entities[1][i].equalsIgnoreCase("dna")) {
202                            mentionName = ClassMentionTypes.DNA;
203                        } else if (entities[1][i].equalsIgnoreCase("rna")) {
204                            mentionName = ClassMentionTypes.RNA;
205                        } else if (entities[1][i].equalsIgnoreCase("cell_line")) {
206                            mentionName = ClassMentionTypes.CELL_LINE;
207                        } else if (entities[1][i].equalsIgnoreCase("cell_type")) {
208                            mentionName = ClassMentionTypes.CELL_TYPE;
209                        } else {
210                            System.err.println("***********   Unrecognized entity type: " + entities[1][i] + "  ***********************");
211                        }
212    
213                        CCPSemanticAnnotation sa = new CCPSemanticAnnotation(jcas);
214                        
215                        // set the annotator
216                        CCPAnnotator ccpAnnotator = new CCPAnnotator(jcas);
217                        if (useBioCreativeModel) {
218                        ccpAnnotator.setAnnotatorID(2);
219                        ccpAnnotator.setLastName("ABNER - BC");
220                        } else {
221                            ccpAnnotator.setAnnotatorID(42);
222                            ccpAnnotator.setLastName("ABNER - NLPBA");
223                        }
224                        
225                        ccpAnnotator.setAffiliation("WISC");
226                        ccpAnnotator.setFirstName("");
227                        
228                        sa.setAnnotator(ccpAnnotator);
229    
230                        // set the annotation set
231                        CCPAnnotationSet ccpAnnotationSet = new CCPAnnotationSet(jcas);
232                        ccpAnnotationSet.setAnnotationSetID(-1);
233                        ccpAnnotationSet.setAnnotationSetName("Default Annotation Set");
234                        ccpAnnotationSet.setAnnotationSetDescription("Default Annotation Set");
235                        FSArray annotationSets = new FSArray(jcas, 1);
236                        annotationSets.set(0, ccpAnnotationSet);
237                        sa.setAnnotationSets(annotationSets);
238    
239                        // set spans
240                        sa.setBegin(Integer.parseInt(span[0]));
241                        sa.setEnd(Integer.parseInt(span[1]));
242                        CCPSpan ccpSpan = new CCPSpan(jcas);
243                        ccpSpan.setSpanStart(Integer.parseInt(span[0]));
244                        ccpSpan.setSpanEnd(Integer.parseInt(span[1]));
245                        FSArray ccpSpans = new FSArray(jcas, 1);
246                        ccpSpans.set(0, ccpSpan);
247                        sa.setSpans(ccpSpans);
248    //                    sa.setDocumentSectionID(documentSectionID);
249    
250                        CCPClassMention ccpClassMention = new CCPClassMention(jcas);
251                        ccpClassMention.setMentionName(mentionName);
252                        ccpClassMention.addToIndexes();
253    
254    //                    /* if it's a protein, then add an ENTREZ_GENE_ID FIELD */
255    //                    if (mentionName.equals(ClassMentionTypes.PROTEIN)) {
256    //                        CCPNonComplexSlotMention ccpNonComplexSlotMention = new CCPNonComplexSlotMention(jcas);
257    //                        ccpNonComplexSlotMention.setMentionName(SlotMentionTypes.PROTEIN_ENTREZGENEID);
258    //                        StringArray slotValues = new StringArray(jcas, 1);
259    //                        slotValues.set(0, "-1");
260    //
261    //                        ccpNonComplexSlotMention.setSlotValues(slotValues);
262    //
263    //                        FSArray ccpSlotMentions = new FSArray(jcas, 1);
264    //                        ccpSlotMentions.set(0, ccpNonComplexSlotMention);
265    //
266    //                        ccpClassMention.setSlotMentions(ccpSlotMentions);
267    //                    }
268    
269                        // Add annotation back-pointer
270                        FSArray ccpTextAnnotations = new FSArray(jcas, 1);
271                        ccpTextAnnotations.set(0, sa);
272                        ccpClassMention.setCcpTextAnnotations(ccpTextAnnotations);
273    
274                        sa.setClassMention(ccpClassMention);
275                        //if (mentionName.equals(ClassMentionTypes.PROTEIN) || mentionName.equals(ClassMentionTypes.CELL_TYPE)) {
276                            sa.addToIndexes();
277                        //}
278                        annotationCount++;
279                    } else { // key already present, so don't do anything
280                        if (DEBUG) {
281                            System.err.println("Preventing duplicate annotation in ABNER.");
282                        }
283                    }
284                } else {
285                    System.err.println("ERROR: NULL SPAN in ABNER ****************************************** Could not find: \"" + entity + "\" in documentText: \"" + docText + "\"");
286                    System.err.println();
287                }
288            }
289    
290            if (annotationCount != entities[0].length) {
291                System.err.println("Error in ABNER_AE: " + entities[0].length + " annotations should have been created, however, only "
292                        + annotationCount++ + " were.");
293    //            throw new AnnotatorProcessException();
294            }
295        }
296    
297        private String[] findSpan(String subStr, String wholeStr) {
298            // create a regular expression for each gene - tok by space, then add
299            // //s? in between tokens
300            // this should work for all genes except those that appear >1 time in
301            // the text
302            // solution, keep track of index from where to start looking for
303            // matches.
304    
305            // returns span[0]: start index; span[1]: end index; span[2]: covered
306            // text
307    
308            String[] span = new String[3];
309            // String escapedChars = "(){}+*?[]^$.\\";
310            String[] escapedChars = { "\\", "(", ")", "{", "}", "+", "*", "?", "[", "]", "^", "$", "." };
311            String escape = "\\";
312            int splitIndex = -1;
313            String tmpStr = "";
314            for (int i = 0; i < escapedChars.length; i++) {
315                if ((splitIndex = subStr.indexOf(escapedChars[i])) > -1) {
316                    int fromIndex = 0;
317                    while (splitIndex > -1) { // check for mulitiple of same
318                        // escapedChar in substr
319                        // insert \ in front of the escaped character
320                        tmpStr = subStr.substring(0, splitIndex) + escape + subStr.substring(splitIndex);
321                        subStr = tmpStr;
322                        fromIndex = splitIndex + 2;
323                        splitIndex = subStr.indexOf(escapedChars[i], fromIndex);
324                    }
325                }
326            }
327    
328            String[] toks = subStr.split(" ");
329            String regex = "";
330            for (int i = 0; i < toks.length; i++) {
331                // if (escapedChars.indexOf(toks[i]) > -1) {
332                // regex += "\\";
333                // }
334                regex += toks[i];
335                if (i < toks.length - 1) {
336                    regex += "\\s*";
337                } else {
338                    regex += "";
339                }
340            }
341    
342            if (DEBUG) {
343                System.out.println("REGEX:" + regex);
344            }
345    
346            Pattern subStrPattern = Pattern.compile(regex);
347            Matcher subStrMatcher = subStrPattern.matcher(wholeStr);
348    
349            if (subStrMatcher.find(startLookingForMatchesAt)) {
350                span[0] = Integer.toString(subStrMatcher.start());
351                span[1] = Integer.toString(subStrMatcher.end());
352                span[2] = subStrMatcher.group();
353                startLookingForMatchesAt = subStrMatcher.end();
354            }
355    
356            return span;
357        }
358    
359       
360    }
```
